# A nucleic acid amplification test‐based strategy does not help inform return to work for healthcare workers with COVID‐19

**DOI:** 10.1111/irv.13000

**Published:** 2022-05-26

**Authors:** Diego R. Hijano, James M. Hoffman, Richard J. Webby, Li Tang, Hana Hakim, Randall T. Hayden, Aditya H. Gaur

**Affiliations:** ^1^ Department of Infectious Diseases St. Jude Children's Research Hospital Memphis Tennessee USA; ^2^ Department of Pharmaceutical Sciences St. Jude Children's Research Hospital Memphis Tennessee USA; ^3^ Department of the Office of Quality and Patient Care St. Jude Children's Research Hospital Memphis Tennessee USA; ^4^ Department of Biostatistics St. Jude Children's Research Hospital Memphis Tennessee USA; ^5^ Department of Pathology St. Jude Children's Research Hospital Memphis Tennessee USA

**Keywords:** antigen test, COVID‐19, healthcare workers, isolation, NAAT, SARS‐CoV‐2

## Abstract

**Objective:**

The objective of this study is to assess the utility of a nucleic acid amplification test‐based approach to shorten isolation of healthcare workers (HCWs) with COVID‐19 in the setting of the highly transmissible omicron variant.

**Methods:**

Between December 24, 2021, and January 5, 2022, HCWs who tested positive for SARS‐CoV‐2 were retested with PCR at least 5 days since onset of symptoms.

**Results:**

Forty‐six sequential fully COVID‐19 vaccinated HCWs who had tested positive for SARS‐CoV‐2 underwent follow‐up testing. All the samples were confirmed as omicron variants and only four (8.7%) were negative in the follow‐up test performed at a median of 6 (range 5–12) since onset of symptoms.

**Conclusions:**

Implementation of a test‐based strategy is logistically challenging, increases costs, and did not lead to shorter isolation in our institution.

## 1 INTRODUCTION

Throughout the pandemic, healthcare institutions had to balance between prevention of COVID‐19 and avoiding staff shortages.^1^ The emergence of the highly transmissible omicron variant associated with a rapid rise in infection irrespective of previous COVID‐19 vaccination status put further stress on the operations of healthcare systems.^2^ In response, the CDC modified their recommendations to shorten isolation periods using follow‐up testing. This test‐based strategy for return to work for healthcare workers (HCWs) who test positive for SARS‐CoV‐2 remains a part of the conventional (work restriction for 10 days but can be 7 if test negative) and contingency recommendations (work restriction for 5 days with/without a negative antigen test or nucleic acid amplification test [NAAT]).^3^ While a symptom‐based strategy is recommended for the public, CDC guidance notes that “if an individual has access to a test and wants to test, the best approach is to use an antigen test towards the end of the 5‐day isolation period.”^4^ CDC guidance appropriately notes that some people may remain positive with NAAT^5^ and while antigen testing is listed as an option, there is no data supporting that antigen tests accurately measure infectivity and can also remain positive after initial infection. We share our experience following the CDC recommended contingency plan with NAAT testing around 5 days from symptom onset or initial test positive (whichever came first) to guide return to work in SARS‐CoV‐2 NAAT‐positive HCWs at a large pediatric specialty hospital providing care for immunocompromised children.

## 2 ETHICAL STATEMENT

The study was deemed exempt research by St Jude's institutional review board with waiver of informed consent.

We implemented the NAAT‐based strategy to inform shorter period of isolation from December 24, 2021, to January 5, 2022, in 46 sequential fully COVID‐19 vaccinated HCWs who had tested positive for SARS‐CoV‐2 (Table 1). In this sample, all the samples were confirmed as omicron variants and only four (8.7%) were negative on the follow‐up test done a median of 6 days (range 5–12 days) from symptom onset. These contemporary findings are consistent with our findings pre‐dating availability of COVID‐19 vaccination, where we had noted a median time to first negative test in NAAT‐positive HCWs of 42 days (range 14–56 days), with testing performed about once a week (unpublished data). Persistent positive PCR tests after recovery from COVID‐19 infection have been well described.^6^

**TABLE 1:** Characteristic of 46 sequential fully vaccinated healthcare workers who had tested positive for SARS‐CoV‐2

	N = 46
Primary vaccine series, number (%)	
Pfizer‐BioNTech	42 (91.3%)
Moderna	2 (4.35%)
Johnson & Johnson	2 (4.35%)
Booster,^a^ number (%)	
Yes	18 (39.13%)
No	28 (60.7%)
Time from booster to COVID‐19 diagnosis (median, range) in days	75 (36–86) days
Time from onset of symptoms to first positive SARS‐CoV‐2 tests (median, range)	1 (1–5) days
Time from COVID‐19 symptoms onset to follow‐up test (median, range) in days	6 (5–12) days
Time from first SARS‐CoV‐2 positive test to follow‐up test (median, range) in days	5 (1–12) days
Follow‐up NAAT, number (%)	
Positive	42 (91.3%)
Negative	4 (8.7%)^b^

^a^ All 18 individuals received homologous booster with Pfizer‐BioNTech.

^b^ All four individuals vaccinated with Pfizer‐BioNTech: Two had received homologous booster, and two had not received a booster dose.

Testing involves time and resources and in our experience did not facilitate a quicker return to work for HCWs providing patient care. In fact, Shenoy *et al* estimated, before vaccines became available, that the test‐based approach recommended early in the pandemic accounted for more than seven additional days of work lost per employee.^7^ Therefore, we moved back to a symptom and time‐based strategy for ending isolation, which is an alternative noted in the CDC guidance for HCWs. However, we chose 7 instead of the 5 days noted in the contingency standards from onset of symptoms or first positive test whichever came first.^4^ While NAAT is qualitative assays, throughout the pandemic, Ct values have been used as a surrogate of viral load and infectiousness. Several scientific entities, such as the Infectious Diseases Society of America, the Association of Molecular Pathology, and the American Association for Clinical Chemistry, have published statements advising against reporting Ct values and using Ct values in medical decision.^8^ CDC suggests that antigen testing in this setting may be preferred to NAAT, given these tests may correlate better with infectiousness.^9^, ^10^ However, a major limitation in all these studies is the use of culture positivity as a surrogate for transmissibility. In addition, Stiefel *et al* found that almost half of HCWs with SARS‐CoV‐2 infection have a positive antigen test 5 days after diagnosis, irrespective of whether they were vaccinated or not further suggesting that the use of follow‐up testing does not lead to shorter isolation in most cases.^11^ While the increased transmissibility of the omicron variant has been noted,^12^, ^13^, ^14^ data defining the specific period of omicron transmissibility remain sparse.^15^ This combined with limited data on the correlation between viral RNA or antigen loads and infectiousness, particularly after the peak of viral replication, and the probability of transmission of infection after 5–7 days from onset of illness appearing low, makes incorporation of a test‐based strategy to determine the period of isolation, of questionable value. A test‐based strategy for return to work inadvertently assigns value to a negative test such that a persistent positive test could get considered an indicator of ongoing infectiousness. In addition, it perpetuates the off‐indication, out‐of‐context use of these tests that were developed for diagnosis of SARS‐CoV‐2 infection and not meant to be used as surrogates of infectiousness.

Emphasis on layers of protection for HCWs must remain, including daily symptom monitoring, universal use of masks, and continued vigilance for workplace transmissions through prompt case investigation and contact tracing to mitigate COVID‐19 in the workplace. If such transmission is detected, it should be assessed in the context of vulnerabilities related to the current, shortened isolation period. Pending transmissibility data to the contrary, symptom and time‐based isolation paradigms without references to incorporating negative test results other than for the immunocompromised or severely ill individuals are the pragmatic path forward at this time.

## AUTHOR CONTRIBUTIONS

**Diego R. Hijano:** Conceptualization; data curation; formal analysis. **James M. Hoffman:** Supervision. **Richard J. Webby:** Supervision. **Li Tang:** Data curation; formal analysis. **Hana Hakim:** Supervision. **Randall T. Hayden:** Resources; supervision; validation. **Aditya H. Gaur:** Supervision; validation.

### PEER REVIEW

The peer review history for this article is available at https://publons.com/publon/10.1111/irv.13000.

## Data Availability

The data that support the findings of this study are available on request from the corresponding author. The data are not publicly available due to privacy or ethical restrictions.
